# Analyzing Time Use by Occupation Area between Weekdays and Weekends by Gender of Parents in Single-Parent Families

**DOI:** 10.1155/2023/8896891

**Published:** 2023-10-03

**Authors:** Woo-Hyuk Jang, Jin-Hyuk Bang

**Affiliations:** Department of Occupational Therapy, College of Health Science, Kangwon National University, Samcheok, Gangwon-do, Republic of Korea

## Abstract

**Introduction:**

Based on the “2019 Living Time Survey” data conducted by the National Statistical Office, this study is aimed at comparing the time usage of single-parent families by classifying the occupation area based on the fourth edition of the occupational therapy practice framework (OTPF) and dividing time use according to weekdays and weekends and by gender.

**Methods:**

After extracting the subjects who were selected belonging to a single-parent family from the original data of the “2019 Living Time Survey,” 404 subjects who were single parents were selected, and the data were analysed. The sample consisted of 134 males and 270 females. The time usage by occupation area during weekdays and weekends and according to the gender of the subjects was checked. By using SPSS 25.0 version for data analysis, the general characteristics and time usage of subjects were presented as frequency analysis and technical statistics, and an independent sample *t*-test was conducted to compare time usage on weekdays and weekends.

**Results:**

Subjects spent more time on “education” and “work” on weekdays, and on weekends, they spent more time on “activities of daily living,” “rest and sleep,” “leisure,” and “social participation.” After classifying the subjects by gender, men spent more time on “work” on weekdays, and on weekends, they spent more time on “activities of daily living,” “rest and sleep,” “leisure,” and “social participation.” Women spent more time on “education” and “work” on weekdays, and on weekends, they spent more time on “rest and sleep,” “leisure,” and “social participation.”

**Conclusion:**

By examining the time usage of single-parent families according to the day of the week, we hope that it will be used as basic data to suggest in which areas they have difficulty managing time, depending on the day of the week, and to establish solutions to insufficient time use due to gender differences.

## 1. Introduction

Modern families are changing into various forms of families, such as dual-income families, childless families, and single-parent families. This is due to the increase in the number of nuclear families as a result of the development of industrialization, changes in gender equality, life views, and women's social advancement [1]. Among them, single-parent families are one of the types of families with a high growth rate [2]. In general, a single-parent family is a family in which one of the parents plays the role of a parent alone due to bereavement or divorce from a spouse [3], and family types are diversifying within a single-parent family, including an increase in the number of rich and unmarried families with only fathers present [4]. Single-parent families experience fatigue in their added roles due to the absence of a spouse, economic poverty, child rearing, and difficulties in housework [5]. Moreover, single-parent families also have difficulty using time because they are responsible for paid labor, child rearing, and housework alone to address economic poverty [6].

Meanwhile, time is limited to 24 hours a day, so if time is used in one activity, time spent on another activity is inevitably reduced [6]. In order to perform the various roles required of an individual within a limited amount of time properly, it is necessary to select the activities necessary for him/her from among various activities and to allocate time and use them accordingly [7]. For adult men and women, time for paid labor is generally used on weekdays and time for housework or leisure on weekends [8]. In this way, paid labor as well as proper balance with household activities, leisure, and education are important factors in improving the quality of life [9]. Furthermore, awareness of using limited time can also affect the quality of life [10]. These characteristics of time use are related to occupation, which is an activity used in daily life to obtain meaning and purpose in life [11].

Therefore, studies were conducted on various subjects to determine the characteristics of time use according to the occupation area [12–18]. First, after examining the characteristics of time use by age group, it was found that, in Korea, time was spent mostly on education among early adolescents, instrumental activities of daily living (IADL) among adults, and rest and sleep among the elderly [12]. Furthermore, it was suggested that with the passage of time, time spent on play, social participation, and education increased among those in their teens and 20s compared to the past, and time spent working on weekdays and spending leisure time on weekends increased among those in their 40s and 50s [13]. Next, after dividing time into balanced and nonbalanced groups for mothers with infant children, it was found that the balanced group spent less time raising children and more time sleeping and leisure than other groups [14]. A comparison of spinal cord injury patients with the general public's time usage showed that spinal cord injury patients spent more time staying at home and on personal maintenance compared to the general public, but less time spent on leisure [15]. Likewise, when comparing time usage among stroke patients and normal adults, it was found that normal adults spent the most time on work in all ages except for those in their 60s, while stroke patients spent the most time on sleep and rest in all ages [16]. After dividing the areas into sleep, daily activities, labor, and leisure activities for the elderly with disabilities and dividing the groups according to the areas they use frequently, there was no significant difference in time satisfaction between the groups. However, it was suggested that the group that spent more time on daily activities felt there was relatively lack of time [17]. Finally, after examining the amount of time used according to the characteristics of the elderly based in welfare centers, both men and women spent the most time resting and sleeping [18]. Subjects afflicted with stroke spend plenty of time on leisure, and the more spouse or cognitive level they have, the more time they spend on various areas [18]. However, previous studies identified the time usage by classifying the occupation area based on the third edition of the occupational therapy practice framework (OTPF) presented in 2014, not the fourth edition presented in 2020 [12, 13, 15, 17, 18], or by classifying the occupation area according to the researcher's arbitrary criteria [14, 16].

Meanwhile, there have been studies examining the use of time among single-parent families [19–21]. First, a study that examined the difference in leisure activities between ordinary families and single-parent families found that ordinary families engage in more leisure activities with their children on weekends than single-parent families, and showed higher life satisfaction [19]. Next, after examining the lack of time according to the gender of single-parent families, it was reported that women felt relatively less time than men though they were not working [20]. Finally, a study that examined how single-parent family parents use their time according to their gender found that men spend less time on housework than women, but more time on paid labor [21]. As such, there have been studies on time use for single-parent families [19–21], but no studies have examined time use by dividing it into systematic occupation areas, and no studies have examined time use for single-parent families by classifying the occupation area based on the fourth edition of the newly published occupational therapy practice framework in 2020.

Therefore, this study is aimed at classifying time usage from the National Statistical Office's “2019 Living Time Survey” data, which was based on the fourth edition of the occupational therapy practice framework (OTPF), and to divide the time use of single-parent families on weekdays and weekends by gender.

## 2. Materials and Methods

### 2.1. Study Subjects

This study used the original data from the “2019 Living Time Survey” conducted by the National Statistical Office. In the data conducted on household members aged 10 or older within 12,435 sample households nationwide, subjects belonging to single-parent families in the category of ‘single parent-parent-grandchild households' were selected. Among them, data of a total of 404 parents in a single-parent family were classified into the day of the week, gender, age, marital status, education level, and economic activity. This study was carried out after the approval of the Kangwon National Institutional Review Board (KWUIRB-2021-07-002-001).

### 2.2. Study Tool

#### 2.2.1. 2019 Time of Life Survey

This study used the time diary of the “2019 Living Time Survey” of the National Statistical Office, which began in 1999 and was conducted nationwide every five years with subjects aged 10 and older nationwide to understand how subjects use their 24 hours. The time diary was divided into weekdays and weekends (Saturday and Sunday), and respondents were asked to fill in the days of the week they responded to and to write about activities that continued for more than five minutes at 10-minute intervals.

### 2.3. Procedure

#### 2.3.1. Classification of Behaviors according to Occupational Area

In the “2019 Living Time Survey” of the National Statistical Office, the behavior of the subjects was classified into “required time,” “duty time,” and “leisure time.” However, in this study, the subject's behavior was reclassified into eight occupation areas and used based on the 4th edition of the occupational therapy practice framework [11]. Behavior classification according to the occupation area is as follows (Table 1).

### 2.4. Statistical Analysis

The data collected in this study were analysed by using SPSS 25.0 version. The general characteristics and time usage of the study subjects were presented through frequency analysis and descriptive statistics. An independent sample *t*-test was conducted to compare time usage according to weekdays and weekends.

## 3. Results

### 3.1. General Characteristics of the Subjects

The number of subjects was 404 in total, with 243 (60.1%) responding on weekdays and 161 (39.9%) responding on weekends. By gender, there were 134 males (33.2%) and 270 females (66.8%) %). In terms of age, 250 (61.9%) were in their 40s, and in terms of marital status, 336 (83.2%) were divorced, 64 (15.8%) were bereaved, and 4 were unmarried (1.0%). In terms of education level, high school graduates accounted for the highest number at 166 (41.1%), while elementary school graduates were the least with 4 (1.0%). As for economic activity, 320 (79.2%) were working. The detailed general characteristics of the study subjects are as follows (Table 2).

### 3.2. Time Usage by Occupation Area according to Weekday and Weekend Classification

Comparing the amount of time used by single-parent family parents by occupation area and according to the classification of weekdays and weekends, it was found that they spend more time on “education” and “work” on weekdays than on weekends, and on weekends, they spend more time on “activities of daily living,” “rest and sleep,” “leisure,” and “social participation.”

Subitems showed significantly higher time usage on weekdays in “out-of-school learning,” “paid work,” and “other work” than on weekends. On the other hand, “eating,” “home management,” “other instrumental activities of daily living,” “sleep,” “cultural and tourism activities,” “leisure activities using media,” and “relationship activities” showed significantly higher time usage on weekends than on weekdays (Table 3).

### 3.3. Time Usage by Occupation Area according to Men's Weekday and Weekend Classification

After comparing men's time usage by occupation area, more time was spent working on weekdays, and more time was spent on daily life activities, rest and sleep, leisure, and social participation on weekends (Table 4).

In the subitems, the amount of time used on weekdays was significantly higher only on paid days than on weekends. On the other hand, eating, home management, sleep, cultural and tourism activities, sports and leisure activities, and leisure activities using media significantly had higher time usage on weekends than on weekdays (Table 4).

### 3.4. Time Usage by Occupation Area according to Women's Weekday and Weekend Classification

After comparing women's time usage by occupation area, more time was spent on education and work on weekdays, and more time was spent on rest and sleep, leisure, and social participation on weekends (Table 5).

Subitems showed significantly higher time usage on weekdays in locomotion, out-of-school learning, paid work, and other work than on weekends. On the other hand, eating, home management, sleep, leisure activities using media, and relationship activities showed significantly higher time usage on weekends than on weekdays (Table 5).

## 4. Discussion

This study attempted to determine the time use of single-parent family parents according to the classification of weekdays and weekends and by gender. To this end, the National Statistical Office's “2019 Living Time Survey” classified the work area based on the fourth edition of the occupational therapy practice framework (OTPF) and analysed the characteristics thereof. Focusing on the results analysed in this study, they are as follows.

First, after examining the amount of time used by occupation area according to the classification of weekdays and weekends, 10.93 minutes were used more for “Activities of daily living” on weekends than on weekdays, and the time for “eating” on weekends increased by 13.64 minutes in the subitems. Usually, on weekdays, people must eat at work for a fixed time, but, on weekends, eating time seems to have increased because they enjoy leisure life such as talking or watching TV at home while eating with their family. These results are consistent with the results of using more meal time on holidays in previous studies, comparing Korean workers' meal time by dividing it into working days and holidays [22]. Moreover, 36.9 minutes more were spent on “home management” on weekends than on weekdays. However, due to the nature of single-parent families who had to cook at home or do pay labor and housework at the same time, they spent more time on “home care” on weekends than on weekdays. The area with the largest change in weekday and weekend time usage was “work,” which decreased by 197.65 minutes on weekends compared to weekdays, followed by weekend “leisure” and “rest and sleep” by 97.74 minutes and 65.62 minutes, respectively. In the case of “leisure” and “rest and sleep,” it seems that enjoying leisure life, rest, and sleep was not possible on weekdays, as the time for “paid work” on weekends has decreased. Leisure activities using media increased the most in leisure, which seems to be intended to recover from fatigue by resting at home through “leisure activities using media,” such as watching TV indoors, while the amount of time spent on “rest and sleep” also seems to have increased. In previous studies, ordinary families chose travel as the most desired leisure activity, while single-parent families prefer rest activities to travel due to economic conditions or heavy labor burdens [19].

Next, after examining the amount of time used by men and women by occupation area according to the classification of weekdays and weekends, both men and women's “eating” increased in “activities of daily living”, but women's time use decreased on weekends compared to men in “personal maintenance.” These results are consistent with previous studies, showing that women usually spend time on “personal maintenance” such as putting on makeup to go out, but less time spent on weekends than on weekdays, such as going to work or leisure activities outdoors [23]. Furthermore, men's locomotion time increased on weekends compared to weekdays, but women's locomotion time decreased significantly on weekends compared to weekdays. These results are thought to be the result of the difference in the way women spend their leisure time, along with the result that women spend more time sleeping on weekends than men. As can be seen from the tabulation of results, among leisure activities, men showed an increase in “cultural and tourism activities,” “sports and leisure activities,” and “leisure activities using media”, while women showed a significant increase in “leisure activities using media.” In other words, even if they spend the same amount of leisure time, men spend more time on leisure that can be enjoyed outdoors, so it is thought that men's locomotion time increases on weekends, and only women's locomotion time decreases significantly on weekends. Both men and women spent more time on leisure during the weekend, while men spent significantly more time on “cultural and tourism activities” and “sports and leisure activities,” and women only increased “leisure activities using media”. This result seems to reflect that men are at an advantage to take their children alone or spend leisure time outdoors than women due to the nature of single-parent families that take their children out for leisure [19]. In terms of “social participation,” both men and women used more time on weekends, but only women showed a significant increase in “relationship activities.” With the formation of a single-parent family, it seems that men have not only interacted with people around them but also in nonface-to-face situations, as men should also increase their social activities with others.

As such, it was found that single-parent families have difficulty balancing their time on weekdays and weekends because they are responsible for paid labor, housework, and child rearing alone, and face difficulties for different reasons, depending on gender. Previous studies have shown that women consistently spend more time on domestic work than men, regardless of whether they are employed or not [20]. This holds true not only for single-parent families but also for ordinary families [24]. These findings suggest that men show relatively less interest in domestic work than women. Thus, there is a clear need to develop and implement educational programs regarding domestic work for men.

The limitations of this study are as follows. Because of the second analysis of the National Statistical Office's original data, it did not match the proportion of the number of people and gender surveyed on weekdays and weekends, the time usage of simultaneous behaviors, such as eating and watching TV at the same time, and the difference in time use compared to ordinary families. However, despite these limitations, the work area was divided based on the 4th edition of the occupational therapy practice framework, and the time usage of single-parent family parents according to the day of the week was also divided by gender. As a result, it is meaningful that it can be used as basic data to suggest in what areas parents of single-parent families have difficulty using time, depending on the day of the week. Furthermore, it can be divided into men and women to establish other solutions based on insufficient time use.

## 5. Conclusions

Parents of single-parent families spent more time on education and work on weekdays than on weekends, and more time on activities of daily living, rest and sleep, leisure, and social participation on weekends.

When looking at the amount of time spent by dividing the gender of single-parent families according to the day of the week, men spent more time working on weekdays, while, on weekends, they spent more time on activities of daily living, rest and sleep, leisure, and social participation. It was found that women spend more time on education and work on weekdays and more time on rest and sleep, leisure, and social participation on weekends.

Based on these results, it was possible to determine the amount of time used by parents of single-parent families according to the day of the week and to point out the differences in time used between genders depending on the day of the week. Since parental time use can affect children's time use, it is suggested that future studies focus on the time use of children in single-parent households. Such research would hopefully lead to the development of effective solutions for the challenges faced by these families.

## Figures and Tables

**Table 1:** Categorization of lifetime based on occupational therapy practice framework-4.

Area	Code	Activities	Code	Activities	Code	Activities
ADLs	12	Meals and snacks	14	Personal maintenance		
IADLs	41	Food preparation	42	Clothes care	43	Cleaning
	44	Management of housing	45	Vehicle care	46	Care of animals and plants
	47	Purchase of items and services	49	Other home management	51	Care for children under 10 years of age
	52	Care for elementary, middle, and high school students over 10 years of age	53	Care for adult families and household members with long-term care needs	54	Care of independent adult families
	73	Religious activities	74	Ritual activities	91 ~98	Locomotion
Health management	13	Personal medical care				
Rest and sleep	11	Sleep	85	Rest		
Education	31	School activities	32	Out-of-school learning		
Work	21	Employed work	22	Self-employment work	23	Unpaid work in family business
	24	Other work	25	Job-seeking activity	26	Purchasing goods related to job
	61	Nonorganization-based volunteer work	62	Organization-based volunteer work	63	Unpaid training
Leisure	81	Leisure activities using media	82	Cultural and tourism activities	83	Sports and leisure sports
	84	Game and play	89	Other leisure		
Social participation	71	Relationship activities	72	Participating activities		

ADLs: activities of daily living; IADLs: instrumental activities of daily living.

**Table 2:** General Characteristics.

Characteristics		Total *N* (%)	
Day of the week	Weekday	243 (60.1)	
	Weekend	161 (39.9)	

		Total *N* (%)	D:E (%:%)
Gender	Male	134 (33.2)	81 : 53 (60.4 : 39.6)
	Female	270 (66.8)	162 : 108 (60.0 : 40.0)

Age	20~29	14 (3.5)	8 : 6 (57.1 : 42.9)
	30~39	76 (18.8)	50 : 26 (65.8 : 34.2)
	40~49	250 (61.9)	153 : 97 (61.2 : 38.8)
	50~59	62 (15.3)	30 : 32 (48.4 : 54.6)
	60~	2 (0.5)	2 : 0 (100 : 0)

Marital status	Single	4 (1.0)	4 : 0 (100 : 0)
	Bereavement	64 (15.8)	41 : 23 (64.1 : 35.9)
	Divorced	336 (83.2)	198 : 138 (58.9 : 41.1)

Education level	Elementary	4 (1.0)	4 : 0 (100 : 0)
	Middle school	20 (5.0)	10 : 10 (50 : 50)
	High school	166 (41.1)	106 : 60 (63.9 : 36.1)
	College	114 (28.2)	68 : 46 (59.6 : 40.4)
	University	84 (20.8)	45 : 39 (53.6 : 46.4)
	Master's degree or higher	16 (4.0)	10 : 6 (62.5 : 37.5)

Job status	Yes	320 (79.2)	190 : 130 (59.4 : 40.6)
	No	84 (20.8)	53 : 31 (63.1 : 36.9)

D: weekday; E: weekend.

**Table 3:** Time usage by occupation area according to day of the week.

Area	Weekday (*n* = 243)	Weekend (*n* = 161)	Weekday-weekend	*t*	*p* value
	Mean ± SD	Mean ± SD			
ADLs	182.18 ± 50.96	193.11 ± 59.32	-10.93	-1.975^∗^	0.049
Eating	102.51 ± 39.34	116.15 ± 49.40	-13.64	-3.077^∗∗^	0.002
Personal maintenance	79.67 ± 34.07	76.96 ± 39.42	2.71	0.736	0.462
IADLs	274.61 ± 141.89	303.23 ± 146.09	-28.62	-1.962	0.050
Locomotion	98.93 ± 62.11	87.08 ± 85.75	11.85	1.609	0.108
Home management	124.53 ± 99.97	161.43 ± 116.66	-36.9	-3.396^∗∗^	0.001
Care of family and members	47.00 ± 68.02	39.01 ± 68.86	7.99	1.15	0.251
Other	4.16 ± 24.59	15.71 ± 50.73	-11.55	-3.053^∗∗^	0.002
Health management	9.05 ± 33.21	5.53 ± 24.49	3.52	1.155	0.249
Personal medical care	9.05 ± 33.21	5.53 ± 24.49	3.52	1.155	0.249
Rest and sleep	474.32 ± 93.01	539.94 ± 122.98	-65.62	-6.094^∗∗∗^	0.000
Rest	13.00 ± 24.84	13.17 ± 25.87	-0.17	-0.064	0.949
Sleep	461.32 ± 93.72	526.77 ± 123.58	-65.45	-6.042^∗∗∗^	0.000
Education	15.39 ± 65.26	0 ± 0	15.39	2.991^∗∗^	0.003
School activities	0.99 ± 15.40	0 ± 0	0.99	0.814	0.416
Out-of-school learning	14.40 ± 61.74	0 ± 0	14.4	2.959^∗∗^	0.003
Work	305.97 ± 219.25	108.32 ± 180.86	197.65	9.495^∗∗∗^	0.000
Paid work	290.74 ± 212.17	102.05 ± 178.32	188.69	9.313^∗∗∗^	0.220
Unpaid work	—	—			
Volunteer work	1.36 ± 13.37	0.06 ± 0.79	1.3	1.228	0.220
Job-seeking activity	0.86 ± 7.90	0.75 ± 9.46	0.11	0.137	0.891
Other	13.00 ± 24.60	5.47 ± 25.35	7.53	2.979^∗∗^	0.003
Leisure	143.50 ± 120.78	241.24 ± 158.50	-97.74	-7.019^∗∗∗^	0.000
Cultural and tourism activities	2.39 ± 14.55	8.82 ± 36.82	-6.43	-2.451^∗^	0.015
Sports and leisure sports	20.86 ± 45.95	23.29 ± 60.53	-2.43	-0.457	0.648
Leisure activities using media	107.41 ± 101.51	187.95 ± 153.88	-80.54	-6.34^∗∗∗^	0.000
Game and play	5.72 ± 23.14	10.25 ± 29.71	-4.53	-1.717	0.087
Other	7.12 ± 30.31	10.93 ± 41.03	-3.81	-1.073	0.284
Social participation	34.98 ± 38.88	48.63 ± 56.83	-13.65	-2.868^∗∗^	0.004
Relationship activities	23.79 ± 33.83	35.34 ± 53.77	-11.55	-2.651^∗∗^	0.008
Participating activities	11.19 ± 17.76	13.29 ± 20.97	-2.1	-1.081	0.280

^∗^ *p* < .05, ^∗∗^*p* < .01, ^∗∗∗^*p* < .001. ADLs: activities of daily living; IADLs: instrumental activities of daily living; SD: standard deviation.

**Table 4:** Time usage by occupation area according to men's day of the week.

Area	Weekday (*n* = 81)	Weekend (*n* = 53)	Weekday-weekend	*t*	*p* value
	Mean ± SD	Mean ± SD			
ADLs	175.56 ± 45.58	196.23 ± 52.92	-20.67	-2.407^∗^	0.017
Eating	105.06 ± 35.85	121.13 ± 40.41	-16.07	-2.412^∗^	0.017
Personal maintenance	70.49 ± 27.29	75.09 ± 36.41	-4.6	-0.835	0.405
IADLs	206.30 ± 121.52	240.38 ± 129.97	-34.08	-1.544	0.125
Locomotion	97.53 ± 59.59	101.32 ± 92.82	-3.79	-0.288	0.774
Home management	73.33 ± 73.98	103.4 ± 88.95	-30.07	-2.122^∗^	0.036
Care of family and members	35.43 ± 53.57	33.77 ± 50.77	1.66	0.179	0.858
Other	0 ± 0	1.89 ± 13.74	-1.89	-1.239	0.218
Health management	10.37±44.68	8.11 ± 36.17	2.26	0.308	0.759
Personal medical care	10.37 ± 44.68	8.11 ± 36.17	2.26	0.308	0.759
Rest and sleep	464.07 ± 76.37	527.36 ± 110.02	-63.29	-3.931^∗∗∗^	0.000
Rest	16.79 ± 32.36	20.75 ± 36.42	-3.96	-0.66	0.511
Sleep	447.28 ± 82.01	506.6 ± 111.2	-59.32	-3.55^∗∗∗^	0.001
Education	2.72 ± 17.18	0 ± 0	2.72	1.15	0.252
School activities	—	—			
Out-of-school learning	2.72 ± 17.18	0 ± 0	2.72	1.15	0.252
Work	415.93 ± 199.30	127.17 ± 196.91	288.76	8.239^∗∗∗^	0.000
Paid work	395.93 ± 190.1	118.68 ± 196.2	277.25	8.151^∗∗∗^	0.000
Unpaid work	—	—			
Volunteer work	—	—			
Job-seeking activity	0.74 ± 6.67	0 ± 0	0.74	0.808	0.421
Other	19.26 ± 30.85	8.49 ± 33.25	10.77	1.916	0.058
Leisure	138.77 ± 126.23	298.68 ± 166.17	-159.91	-6.317^∗∗∗^	0.000
Cultural and tourism activities	0.62 ± 5.56	17.36 ± 55.48	-16.74	-2.701^∗∗^	0.008
Sports and leisure activities	18.4 ± 50.98	37.17 ± 53.9	-18.77	-2.038^∗^	0.044
Leisure activities using media	102.59 ± 104.7	206.04 ± 172.75	-103.45	-4.316^∗∗∗^	0.000
Game and play	8.02 ± 31.44	13.58 ± 34.31	-5.56	-0.965	0.336
Other	9.14 ± 30.13	24.53 ± 59.31	-15.39	-1.98^∗^	0.050
Social participation	26.30 ± 27.41	42.08 ± 55.17	-15.78	-2.196^∗^	0.030
Relationship activities	15.68 ± 22.69	26.98 ± 51.87	-11.3	-1.727	0.086
Participating activities	10.62 ± 16.98	15.09 ± 22.41	-4.47	-1.313	0.192

^∗^ *p* < 0.05, ^∗∗^*p* < 0.01, ^∗∗∗^*p* < 0.001. ADLs: activities of daily living; IADLs: instrumental activities of daily living; SD: standard deviation.

**Table 5:** Time usage by occupation area according to women's day of the week.

Area	Weekday (*n* = 162)	Weekend (*n* = 108)	Weekday-weekend	*t*	*p* value
	Mean ± SD	Mean ± SD			
ADLs	185.49 ± 53.28	191.57 ± 62.40	-6.08	-0.857	0.392
Eating	101.23 ± 41.02	113.70 ± 53.26	-12.47	-2.168^∗^	0.031
Personal maintenance	84.26 ± 36.22	77.87 ± 40.95	6.39	1.347	0.179
IADLs	308.77 ± 139.29	334.07 ± 144.18	-25.31	-1.442	0.150
Locomotion	99.63 ± 63.50	80.09 ± 81.60	19.54	2.206^∗^	0.028
Home management	150.12 ± 101.61	189.91 ± 118.40	-39.78	-2.948^∗∗^	0.003
Care of family and members	52.78 ± 73.66	41.57 ± 76.27	11.20	1.207	0.228
Other	6.23 ± 29.93	22.50 ± 60.13	-16.27	-2.941^∗∗^	0.004
Health management	8.40 ± 25.78	4.26 ± 16.01	4.14	1.487	0.138
Personal medical care	8.40 ± 25.78	4.26 ± 16.01	4.14	1.487	0.138
Rest and sleep	479.44 ± 100.13	546.11 ± 128.90	-66.67	-4.77^∗∗∗^	0.000
Rest	11.11 ± 19.91	9.44 ± 17.71	1.67	0.704	0.482
Sleep	468.33 ± 98.56	536.67 ± 128.56	-68.33	-4.933^∗∗∗^	0.000
Education	21.73 ± 78.32	0.00 ± 0.00	21.73	2.881^∗∗^	0.004
School activities	1.48 ± 18.86	0.00 ± 0.00	1.48	0.816	0.415
Out-of-school learning	20.25 ± 74.03	0.00 ± 0.00	20.25	2.84^∗∗^	0.005
Work	250.99 ± 208.30	99.07 ± 172.65	151.91	6.276^∗∗∗^	0.000
Paid work	238.15 ± 203.37	93.89 ± 169.22	144.26	6.097^∗∗∗^	0.000
Unpaid work	—	—			
Volunteer work	2.04-16.35	0.09-0.96	1.94	1.234	0.218
Job-seeking activity	0.93 ± 8.47	1.11 ± 11.55	-0.19	-0.152	0.879
Other	9.88 ± 20.19	3.98 ± 20.41	5.90	2.34^∗^	0.020
Leisure	145.86 ± 118.30	213.06 ± 147.35	-67.19	-4.139^∗∗∗^	0.000
Cultural and tourism activities	3.27 ± 17.33	4.63 ± 21.85	-1.36	-0.567	0.571
Sports and leisure activities	22.10 ± 43.33	16.48 ± 62.64	5.62	0.871	0.384
Leisure activities using media	109.81 ± 100.13	179.07 ± 143.75	-69.26	-4.667^∗∗∗^	0.000
Game and play	4.57 ± 17.59	8.61 ± 27.19	-4.04	-1.484	0.139
Other	6.11 ± 30.45	4.26 ± 25.91	1.85	0.519	0.604
Social participation	39.32 ± 42.92	51.85 ± 57.60	-12.53	-2.046^∗^	0.042
Relationship activities	27.84 ± 37.61	39.44 ± 54.44	-11.60	-2.072^∗^	0.039
Participating activities	11.48 ± 18.19	12.41 ± 20.27	-0.93	-0.391	0.696

^∗^ *p* < 0.05, ^∗∗^*p* < 0.01, ^∗∗∗^*p* < 0.001. ADLs: activities of daily living; IADLs: instrumental activities of daily living; SD: standard deviation.

## Data Availability

The (2019 Living Time Survey) data used to support the findings of this study have been deposited in the (Statistics Korea) repository (https://kostat.go.kr/anse/).
